# Why working from home varies across countries and people

**DOI:** 10.1073/pnas.2529036122

**Published:** 2025-12-15

**Authors:** Pablo Zarate, Jose Maria Barrero, Nicholas Bloom, Steven J. Davis, Mathias Dolls, Cevat Giray Aksoy

**Affiliations:** ^a^Department of Economics, Princeton University, Princeton, NJ 08540; ^b^Business School, Instituto Tecnológico Autónomo de México, Mexico City 10700, Mexico; ^c^Department of Economics, Stanford University, Palo Alto, CA 94305; ^d^Hoover Institution at Stanford University, Palo Alto, CA 94305; ^e^Center for Macroeconomics and Surveys, ifo Institute, Munich 81679, Germany; ^f^Office of the Chief Economist, European Bank for Reconstruction and Development and Department of Political Economy, King’s College London, London E14 4BG, United Kingdom

**Keywords:** remote work, culture, global survey

## Abstract

We investigate why work from home (WFH) rates vary so widely across countries using the Global Survey of Working Arrangements (G-SWA). Conducted in late 2024 and early 2025, G-SWA is the only harmonized international survey of remote work, covering 14,427 full-time, college-educated workers across 37 countries. Our analysis finds that cultural individualism accounts for 29 percent of the cross-country variation in WFH rates—more than any other single factor. Industry structure, population density, and economic development account for smaller shares of the cross-country variation. We conclude with brief remarks on the broader role of culture in shaping the future of work and its implications for labor market policy.

The COVID-19 pandemic precipitated a rapid and widespread reorganization of work, with millions of employees transitioning to remote arrangements in a matter of weeks. In the United States, the share of workdays performed from home surged from 7% in 2019 to nearly 60% in spring 2020, before stabilizing at around 25 to 30% by 2023 (1). Similar shifts occurred in many other countries. While past transformations in work (such as the move from farms to factories and factories to offices) unfolded over decades, the work-from-home revolution took place in weeks. Unlike many pandemic-era changes, this one has endured (2).

Despite the scale of the shift, we know little about why some countries have embraced WFH more than others. English-speaking and Northern European countries report substantially higher rates of remote work than much of Asia or Latin America (3, 4), reflecting differences in institutions, technology, and culture. Yet, until recently, the lack of comparable international data made it difficult to systematically assess these determinants.

To address this issue, we present evidence from our own Global Survey of Working Arrangements (G-SWA)—the only recurring, globally harmonized survey of remote work. Its latest wave (Nov 2024–Feb 2025) surveyed 14,427 full-time, college-educated workers across 37 countries, using samples stratified by gender, age, and education. The results reveal striking international differences: In some countries, remote work is now widespread, while in others, it remains relatively uncommon. We use the G-SWA data to assess the structural and cultural forces behind these patterns.

Understanding what shapes remote work adoption across countries is crucial, given its far-reaching implications for individuals, firms, and economies. Evidence on productivity effects is mixed—positive in some contexts (5–7), negative in others (8, 9). Remote work also broadens labor force participation, particularly for groups that value flexibility—such as parents with young children, caregivers, and individuals with disabilities (4, 10, 11). At the macro level, it is transforming urban economies by reshaping real estate markets, commuting patterns, and wage-setting norms (12–15).

## Cross-Country Variation

Our data reveal that rates of work from home (WFH) continue to vary widely across countries. In the 2024–25 wave of the G-SWA, college-educated employees in English-speaking countries (such as the United States, Canada, the United Kingdom, and Ireland) report an average of 1.3 to 1.9 WFH days per week. In contrast, employees in many East Asian countries, including some of the region’s most advanced economies, typically report less than one WFH day per week. Most European and Latin American countries fall in between. Notably, these patterns are stable over time: While the overall prevalence of remote work fell from peaks in 2020, country rankings have changed little since 2023 (2).

What accounts for these differences in WFH rates? A natural starting point is economic structure. Early in the pandemic, researchers estimated that roughly 37% of US jobs could, in principle, be performed entirely from home (16), and many others could be performed partly at home. National economies with high shares of desk-based “knowledge” jobs and strong digital infrastructure are expected to have higher WFH rates. In contrast, economies dominated by manufacturing, retail, or agriculture offer relatively few remote-suitable jobs. On that basis, nations with sizable white-collar sectors and fast, reliable internet might be expected to have higher WFH rates. But nearly five years after the pandemic struck, this explanation is clearly incomplete at best. Several East Asian countries, often with world-class digital infrastructure, still have low levels of remote work. Thus, it appears that factors beyond job characteristics, economic development, and digital infrastructure also shape the extent to which countries embrace working from home.

## Results and Discussion

### The Role of Individualism.

Compared to traditional work practices, remote and hybrid working arrangements involve less in-person oversight by managers, fewer in-person encounters with coworkers, and more scope for personal autonomy in executing work-related tasks. These observations suggest that societies with a strong orientation toward individualism will be more hospitable to WFH. Conversely, societies that are more collectivist in orientation (placing greater value on hierarchy, group cohesion, and face-to-face interactions) will be less hospitable to WFH. Thus, we hypothesize that societies that score highly on individual freedom and self-reliance, as captured by Geert Hofstede’s index of individualism (17), will exhibit higher WFH rates. The review by Alesina and Giuliano (18) among others establishes individualism as an important cultural determinant of economic and institutional outcomes, such as innovation and labor market arrangements (19, 20).

**Fig. 1.**
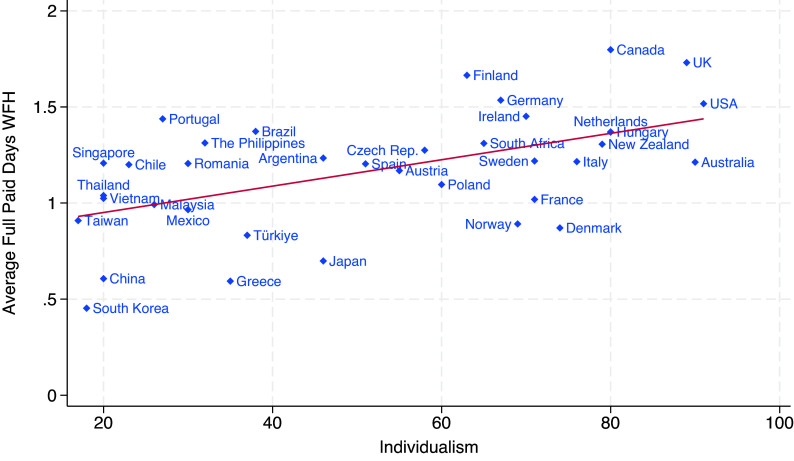
More Individualistic Countries Work from Home More. Note: The figure displays the country-specific values of Individualism and the average paid days per week of WFH among full-time college-educated employees. The correlation between the two variables is of 0.539. Source: Authors’ analysis of G-SWA data, 2024–25 wave.

To assess this hypothesis, we begin by examining the simple cross-country relationship between individualism and remote work. Fig. 1 plots each country’s average number of WFH days per week against its individualism score. The correlation is strong and positive (0.539), suggesting that countries with more individualistic cultures tend to have higher WFH rates. The magnitude is also noteworthy: moving from the 10th percentile (China) to the 90th percentile (Netherlands) in the distribution of individualism index values involves an increase of 0.47 WFH days per week, which equals 38 percent of the average WFH rate across countries.

Next, we fit a multivariate regression model to data for 37 countries from the latest G-SWA wave. We consider six covariates: Hofstede’s index of individualism, the share of jobs suitable for WFH, real GDP per capita, weighted population density, average commute time, and the cumulative stringency of government-mandated lockdowns. We compute the average commute time from the self-reported round-trip commute time to and back from work in our survey. We compute the WFH-suitable job share as the weighted average of industry-level WFH propensities, with weights given by the industry mix of employment in the country (“Industry Mix”). Propensities equal WFH rates in US data from the Survey of Working Arrangements and Attitudes. Real GDP per capita controls for the country’s overall level of economic development. We control for population density using measures from Edwards et al. (21), given prior evidence that WFH rates rise with density (1). Finally, stricter and longer government restrictions on commercial and social activity during the pandemic encouraged more experimentation with, and more learning by doing in, remote work. That could lead to persistently higher WFH rates, other things equal. Accordingly, we measure “Lockdown Stringency” following Aksoy et al. (4). For ease of comparison, we standardize all covariates to have mean zero and unit SD.

**Fig. 2.**
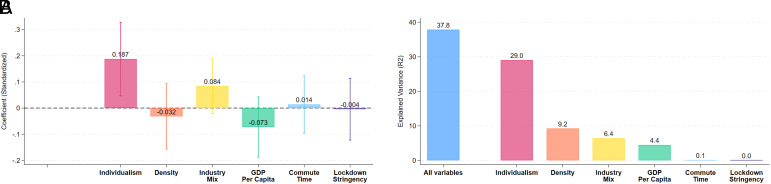
Cultural Individualism Is the Most Important Explanator of Cross-Country Variation in WFH. (*A*) Coefficient estimates on standardized variables in a cross-country regression. (*B*) Variance Explained (R^2^) by Each Predictor and the Full Model. Note: (*A*) reports coefficient estimates in a cross-country multivariate regression of the average paid days WFH on six covariates and a constant. We standardize each covariate to have null mean and variance of one. (*B*) displays the model fit values (R-squared) when considering all six covariates and each one individually.

Panel *A* of Fig. 2 reports the estimated association of each factor when included jointly in the model. The results reinforce the central role of cultural forces: Individualism has the largest and most statistically significant association with WFH intensity. This finding is consistent with a recent paper (22) that builds on an earlier working paper version of our study and shows that individualism helps explain differences in WFH intensity among immigrants from diverse cultural backgrounds residing in the same country. Our paper extends this evidence across countries using direct measures of remote work (rather than proxies) from unique survey data covering developed and developing economies. Our results reveal a robust and systematic relationship that persists even after accounting for a wide range of economic, occupational, and technological factors. Together, these findings highlight the importance of cultural values as a key determinant of how societies adapt to new ways of working.

Looking at other factors, the industry mix is only marginally significant, and its coefficient is about half as large. The other covariates are not statistically significant. In additional analyses, we also considered housing conditions (that is, the share of single-family housing in the total housing stock) as an explanatory variable, but the point estimates were very small and statistically insignificant, and are therefore not reported here. Importantly, when we compute country-level WFH averages separately by gender and re-estimate the model, individualism continues to predict higher levels of remote work for both men and women.

*How much of the international variation in remote work do these factors explain?*

Panel B of Fig. 2 shows that, together, the six variables account for approximately 38% of the cross-country variance in WFH adoption. Notably, individualism alone explains around 29% of this variation in a univariate regression—far more than any other single factor. In fact, the combined explanatory power of the remaining variables is still smaller than that of individualism alone. Put simply, cultural forces appear to exert a powerful influence on the extent to which countries embrace WFH.

## Discussion

Using unique, harmonized survey data from both developed and developing economies, our study provides evidence that individualism is an important predictor of national WFH rates. This finding remains robust after accounting for economic, occupational, and technological factors, underscoring culture’s role as a key determinant of how countries integrate remote work into their labor markets. As remote and hybrid arrangements become more entrenched, policymakers and firms face the challenge of leveraging their benefits while mitigating their risks, including weakened social capital and uneven urban impacts. Taken together, our findings highlight that the future of work will be shaped not only by technology and policy but also by cultural forces that influence how individuals and organizations balance autonomy, trust, and coordination.

## Materials and Methods

### Data and Sample.

We analyze data from the G-SWA Wave 4, conducted between November 2024 and February 2025. The G-SWA is an international survey administered to adult workers via professional survey firms in each country. Wave 4 covers 40 countries, including the United States, Canada, the United Kingdom, dozens of European and Asian economies, as well as a selection of Latin American and African countries. To focus on jobs with WFH potential, the analysis sample targets respondents who are college-educated full-time employees aged 20 to 64. National samples are constructed to be broadly representative of the college-graduate workforce in each country with respect to age, gender, and other demographics (quota sampling is used to ensure balance).

Ethics approval was granted by the Chair of the NBER Institutional Review Board (IRB Ref#24_128), and informed consent was obtained from all participants.

## Supplementary Material

Appendix 01 (PDF)

## Data Availability

The data and replication package (including survey questionnaire) is available in the Harvard Dataverse, which can be accessed through this link: https://doi.org/10.7910/DVN/RPSPPS (23). Survey data have been deposited in Harvard Dataverse (Will be posted if accepted). Survey data have been deposited in Harvard Dataverse (Will be posted if accepted). Study data are included in the article and/or *SI Appendix*.
